# Pyrazinamide resistance-conferring mutations in *pncA* and the transmission of multidrug resistant TB in Georgia

**DOI:** 10.1186/s12879-017-2594-3

**Published:** 2017-07-12

**Authors:** Sarah Sengstake, Indra L Bergval, Anja R Schuitema, Jessica L de Beer, Jody Phelan, Rina de Zwaan, Taane G Clark, Dick van Soolingen, Richard M Anthony

**Affiliations:** 1 Royal Tropical Institute, KIT Biomedical Research, Meibergdreef 39, 1105 AZ, Amsterdam, The Netherlands; 2 Unit of Mycobacteriology, Institute of Tropical Medicine Antwerp, Nationalestraat 155, 2000 Antwerp, Belgium; 3 Centre for Zoonoses and Environmental Microbiology, Centre for Infectious Disease Control (CIb), National Institute for Public Health and the Environment (RIVM), PO Box 1, 3720 BA, Bilthoven, The Netherlands; 4 Mycobacteria Diagnostic Laboratory for Bacteriology and Parasitology (BPD) Center for Infectious Disease Research, Diagnostics and Perinatal Screening (IDS) National Institute for Public Health and the Environment (RIVM), P.O. Box 1, 3720 BA, Bilthoven, The Netherlands; 5 Department of Pathogen Molecular Biology, Faculty of Infectious and Tropical Diseases, London School of Hygiene & Tropical Medicine, Keppel Street, WC1E 7HT, London, UK; 6 Department of Infectious Disease Epidemiology, Faculty of Epidemiology and Population Health, London School of Hygiene & Tropical Medicine, Keppel Street, WC1E 7HT, London, UK

**Keywords:** Mycobacterium tuberculosis, Pyrazinamide, Transmission, Drug resistance, pncA

## Abstract

**Background:**

The ongoing epidemic of multidrug-resistant tuberculosis (MDR-TB) in Georgia highlights the need for more effective control strategies. A new regimen to treat MDR-TB that includes pyrazinamide (PZA) is currently being evaluated and PZA resistance status will largely influence the success of current and future treatment strategies. PZA susceptibility testing was not routinely performed at the National Reference Laboratory (NRL) in Tbilisi between 2010 and September 2015. We here provide a first insight into the prevalence of PZA resistant TB in this region.

**Methods:**

Phenotypic susceptibility to PZA was determined in a convenience collection of well-characterised TB patient isolates collected at the NRL in Tbilisi between 2012 and 2013. In addition, the *pncA* gene was sequenced and whole genome sequencing was performed on two isolates.

**Results:**

Out of 57 isolates tested 33 (57.9%) showed phenotypic drug resistance to PZA and had a single *pncA* mutation. All of these 33 isolates were MDR-TB strains. *pncA* mutations were absent in all but one of the 24 PZA susceptible isolate. In total we found 18 polymorphisms in the *pncA* gene. From the two major MDR-TB clusters represented (94–32 and 100–32), 10 of 15, 67.0% and 13 of 14, 93.0% strains, respectively were PZA resistant. We also identified a member of the potentially highly transmissive clade A strain carrying the characteristic I6L substitution in PncA. Another strain with the same MLVA type as the clade A strain acquired a different mutation in *pncA* and was genetically more distantly related suggesting that different branches of this particular lineage have been introduced into this region.

**Conclusion:**

In this high MDR-TB setting more than half of the tested MDR-TB isolates were resistant to PZA. As PZA is part of current and planned MDR-TB treatment regimens this is alarming and deserves the attention of health authorities. Based on our typing and sequence analysis results we conclude that PZA resistance is the result of primary transmission as well as acquisition within the patient and recommend prospective genotyping and PZA resistance testing in high MDR-TB settings. This is of utmost importance in order to preserve bacterial susceptibility to PZA to help protect (new) second line drugs in PZA containing regimens.

## Background

Pyrazinamide (PZA) has been part of the standard first line TB treatment since the 1980s. Adding PZA to the treatment regimen had a significant effect on the duration of treatment needed to cure TB (six instead of nine months) (reviewed in [1, 2]). Its unique activity against metabolically inactive, slow growing bacteria make PZA not only an invaluable component of the current first line treatment regimen, but also attractive for inclusion in novel treatment regimens for susceptible TB [3, 4] as well as for the treatment of drug resistant TB [5]. In high MDR-TB settings worse treatment outcomes are observed if the infecting strain is resistant to PZA, especially when resistance to fluoroquinolones is also present [6, 7]. It is therefore of utmost importance to preserve bacterial susceptibility to PZA in order to ensure the effective treatment of (MDR-)TB.

Drug susceptibility testing (DST) for PZA is not routinely used to guide (first and second line) anti-TB therapy. Due to the technical complexity of DST for PZA the results of phenotypic tests assessing the susceptibility of TB bacteria to PZA are not always considered to accurately reflect the resistance status of the bacteria [8]. The MGIT system is the most standardised and reliable method to perform DST for PZA [1, 9] and is currently the only commercially available phenotypic test to investigate PZA susceptibility. Here, clinical specimens are cultured in the presence of PZA under acidic conditions mimicking the in vivo conditions under which pyrazinoic acid (the activated drug) is active [1]. These issues have stimulated efforts to inform the design of molecular assays for the detection of PZA resistance [10, 11] but these methods are not currently widely applied.

For the reasons explained above the WHO recommends inclusion of PZA in the treatment regimen regardless of the results of PZA susceptibility testing [12]. Consequently, PZA susceptibility testing is often not prioritised. As a result there is often only limited data available on PZA resistance trends and epidemiology.

Resistance to PZA is largely conferred by mutations in the *pncA* gene, which encodes the pyrazinamidase enzyme that activates the drug [10, 13]. Studies that have assessed PZA resistance typically report about 40–50% in primary MDR-TB [14–17] and more than 90% in XDR-TB isolates (TBNET) [18]. Alignment of the protein sequence of PZA from various bacterial species revealed three conserved regions [19], moreover three mutational hot spot regions in *pncA* have been proposed [20]. However recent systematic reviews comparing large data sets do not support extensive clustering of mutations in *pncA* [10, 11] but rather define large numbers of high confidence mutations that when combined can identify molecular PZA resistance with an accuracy between 89.5 and 98.9% [10]. The observed diversity of *pncA* mutations found in clinical PZA resistant strains is in strong contrast to the limited spectrum of mutations conferring resistance to isoniazid and rifampicin [21, 22]. Clusters of PZA resistant strains with identical *pncA* mutations have been reported but so far have been geographically restricted [23–27]. The presence of clustered strains all having the same rifampicin- and isoniazid-resistance conferring mutations but dispersed *pncA* mutations has stimulated the idea that PZA resistance mutations are acquired post-MDR and that the presence of PZA conferring mutations could represent a bottleneck for transmission [28].

The National Tuberculosis Reference centre in Tbilisi, Georgia, suspended DST for PZA in 2010 *(*M. Akhalaia/ N. Tukvadze, *personal communication*) but have resumed DST for PZA in September 2015 (N. Bablishvili, *personal communication).* PZA remains an important component of the standard first line and second line treatment regimen in this setting. Georgia is recognised as one of the high-burden MDR-TB countries with 16.6% MDR-TB patients reported among all laboratory confirmed pulmonary TB patients in 2014 [29]. Although the percentage of new MDR-TB cases remains constant the percentage of retreatment MDR-TB cases is increasing. The incidence and prevalence of PZA resistance is unknown in this setting. Here we report phenotypic susceptibility and molecular resistance to PZA in a convenience collection of primary MDR-TB isolates from Georgia collected between 2012 and 2013.

## Material and methods

### Patient samples

Samples were part of a larger study of 399 clinical isolates of newly diagnosed patients with pulmonary TB collected between 2012 and 2013 at the NCTLD in Tbilisi, Georgia [30]. Of the 399 samples 67 were subjected to more detailed genetic investigation. These samples were selected to reflect the high proportion of MDR-TB strains and strains of the Beijing genotype previously found in this collection. Coletsos slopes of all 67 samples were subjected to PZA DST and Sanger sequencing of the *pncA* gene. Interpretable PZA DST results were obtained for 57 strains. Two strains were subjected to WGS.

The study was exempt from the approval of the Local Ethics Committee of the National Center for Tuberculosis and Lung Diseases (50 Maruashvili St, 0101 Tbilisi, Georgia) and Informed Consent was not required as the patient information used was anonymised before linking to the results of the analysis of the bacterial cultures and could not be linked back to individual patients.

### Drug susceptibility testing

Drug susceptibility testing for PZA was performed according to the manufacturer’s instructions in the BD BACTEC MGIT 960 system. Bacteria from an inoculum of at least one day positive and a maximum of 5 day positive, according to the BACTEC MGIT, were exposed to 100 ul/ml PZA in an pH 5.9 environment. A strain was determined resistant if at the moment when the growth control (GC) reaches 400 growth units (GU) the PZA tube had reached at least a 100 growth units.

### Sanger sequencing

Of the 57 samples tested by PZA DST 33 samples that were PZA resistant by DST and all 24 strains that were susceptible had their *pncA* gene amplified and sequenced. The *pncA* gene was sequenced using pncA_1F: GGC CGC GAT GAC ACC TCT, pncA_1R: GCC GCA GCC AAT TCA GCA GT, pncA_2F: CGA AGC GGC GGA CTA CCA TCA CG and pncA_2R: CCC CAC CTG CGG CTG CGA ACC and the Sanger sequencing technology (BaseClear, Leiden, The Netherlands). Sequences were analysed using BioNumerics (Applied Maths).

### Whole genome sequencing

CTAB purified DNA was prepared from isolates 12-17889 and 12-15893 and whole genome sequences were prepared at GATC Biotech (GATC, Konstanz, Germany) on a Illumina HiSeq 2500 device using paired-end reads of 2x150bp and minimum coverage of 400 reads in the core genome. The FASTQ files were mapped to the H37Rv reference genome using *bwa-mem* software. From the resulting alignments, *SAMtools* and *GATK* software suites (default parameter settings) were used to call SNPs and small indels, and the intersection of variants between the methods retained. A full description of the pipeline is described elsewhere [31, 32]). The strain 12–17,889 was shared with N. Casali (*Devision of Infectious Diseases,* Imperial College London). Sequence analysis as well as phylogenetic analysis of strain 12-17889 was performed as previously described [26].

### MIRU-VNTR typing

All 57 samples were typed by 24-locus VNTR [33] either at the RIVM or by Genoscreen (Lille, France). MLVA MtbC 15–9 types were assigned using the MIRU-VNTR*plus* web application [34] [35] based on the MIRU-VNTR*plus* database. A cluster was defined as two or more strains having the same MLVA type.

## Results

**Fig. 1:**
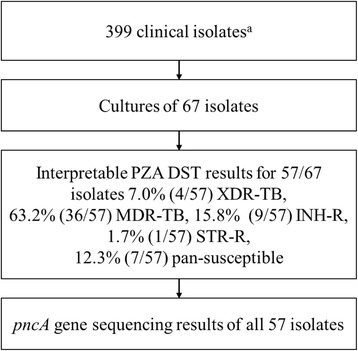
Flowchart of available information. XDR = extensively drug resistant, MDR = multidrug resistant tuberculosis, INH-R = isoniazid monoresistant, STR-R = streptomycin mono resistant. a = [30]

PZA DST results were obtained for 57 strains (Fig. 1). Of these 57.9% (33/57) were resistant and 42.1% (24/57) were susceptible to PZA. Of the tested 57 strains 63.1% (36/57) were MDR-TB and 7.0% (4/57) were XDR-TB by DST (Table 1). These 40 M(X)DR-TB samples represent 54.0% (40/74) of all the M(X)DR-TB isolates identified in a the larger collection of 399 strains [30]. Of the remaining 17 strains nine were monoresistant to isoniazid and seven were pan-susceptible and one strain was streptomycin mono-resistant. Of all 57 strains 24 (24/57, 42.1%) were from patients with a previous TB treatment history and 33 (33/57, 57.9%) were from newly diagnosed patients. Of the 24 retreatment cases 17 (17/24, 70%) were PZA resistant and of the 33 new patients 16 (16/33, 48%) were PZA resistant.

**Table 1:** Summary of *pncA* polymorphisms of all 57 strains with interpretable PZA DST results

Strain	Nucleotide	Substitution	DST+[Z]^a^	MTB lineage^b^	MLVA 15–9 type	Previously reported	Category^c^
12-14129	T14C	Ile5Thr	MDR+[R]	Haarlem	15898-15	No	−
12–17,889	A16C	Ile 6 Leu	MDR+[S]	Beijing K1	1065–32	Yes [10]	C
12–15893	T20C	Val7Ala	XDR+[R]	Beijing K1	1065–32	Yes [11, 37]	−
12–16359	T40C	Cys14Arg	MDR+[R]	Beijing V+/CHIN+	?-32	Yes [10, 11, 37, 46]	A
13–1786	G41A	Cys14Tyr	XDR+[R]	Beijing V+/CHIN+	100–32	Yes [37, 46]	−
12–16734	A146C	Asp49Ala	MDR+[R]	Beijing V+/CHIN+	100–32	Yes [10, 37, 46]	A
12–16077	A188C	Asp63Ala	MDR+[R]	Beijing V+/CHIN+	100–32	Yes [10, 37]	D
12–16119	G204 T	Trp68Cys	MDR+[R]	Haarlem	2943–31	Yes [10]	A
12–17593	C211T	His71Tyr	MDR+[R]	Beijing V+/CHIN+	100–32	Yes [10, 11, 37, 46]	A
12–14879 12–17704 12–19027 12–19131	A212C	His71Pro	MDR+[R]	Beijing K1	94–32	Yes [37]	−
13–774	A212G	His71Arg	MDR+[R]	Beijing V+/CHIN+	1047–32	Yes [10, 11, 37, 46]	A
12–15175 12–17995 12–18248 12–16269	A212G	His71Arg	M(X)DR+[R]	Beijing K1	94–32	See above	See above
12–13963	T254G	Leu85Arg	MDR+[R]	Beijing K1	407–32	Yes [10, 11, 37, 46]	A
12–14551	G356 T	Trp119Leu	MDR+[R]	Beijing K1	94–32	Yes [37]	−
12–17975	T389G	Val130Gly	MDR+[R]	Beijing V+/CHIN+	9375–32	Yes [10, 11, 37, 46]	A
12–16180	T412C	Cys138Arg	XDR+[R]	Beijing V+/CHIN+	100–32	Yes [10, 11, 37, 46]	A
12–17047	A422C	Gln141Pro	MDR+[R]	Beijing V+/CHIN+	100–135	Yes [10, 11, 37, 46]	A
12–18057 12–17993 12–16295 12–15251 12–18055 12–18893 12–19128	A422C	Gln141Pro	MDR+[R]	Beijing V+/CHIN+	100–32	See above	See above
12–17736	A422C	Gln141Pro	MDR+[R]	Beijing V+/CHIN+	9368–32	See above	See above
12–16850	A422C	Gln141Pro	MDR+[R]	Beijing V+/CHIN+	other	See above	See above
12–14180	C425T	Thr142Met	MDR+[R]	Beijing K1	94–32	Yes [10, 11, 37, 46]	A
12–18166	T448:G	Insertion → frameshift	MDR+[R]	Beijing V+/CHIN+	100–32	No	−
13–2219	wildtype	−	MDR+[S]	LAM	8013–52	−	−
12–17795	wildtype	−	mono-INH+[S]	LAM	843–52	−	−
12–16706	wildtype	−	pan-susceptible + [S]	Beijing K1	94–32	−	−
12–16496 12–15156	wildtype	−	mono-INH+[S]	Beijing V+/CHIN+	94–32	−	−
12–16505	wildtype	−	mono-INH+[S]	Beijing V+/CHIN+	407–32	−	−
12–17231	wildtype	−	MDR+[S]	Beijing V+/CHIN+	94–32	−	−
12–15460	wildtype	−	pan-susceptible + [S]	Beijing V+/CHIN+	94–32	−	−
13–56	wildtype	−	MDR-TB+[S]	Beijing V+/CHIN+	100–32	−	−
12–18360	wildtype	−	MDR-TB+[S]	Beijing V-	14852–32	−	−
12–19069	wildtype	−	MDR-TB+[S]	Euro-American	163–15	−	−
12–18493	wildtype	−	MDR-TB+[S]	Beijing K1	94–32	−	−
12–15155	wildtype	−	INHR+[S]	Euro-American	?-15	−	−
12–13700	wildtype	−	INHR+[S]	Haarlem	?-31	−	−
13–2072	wildtype	−	INHR+[S]	Euro-American	12411–15	−	−
13–421	wildtype	−	INHR+[S]	Haarlem	7361–31	−	−
12–18490	wildtype	−	INHR+[S]	Euro-American	768–26	−	−
13–172	wildtype	−	SR+[S]	Beijing V+/CHIN+	other	−	−
12–15239	wildtype	−	pan-susceptible + [S]	X	?-15	−	−
12–15737	wildtype	−	pan-susceptible + [S]	CAS	1059–25	−	−
12–16196	wildtype	−	pan-susceptible + [S]	Beijing SA−/CHIN-	785–32	−	−
12–18942	wildtype	−	pan-susceptible + [S]	Beijing K1	other	−	−
13–1	wildtype	−	pan-susceptible + [S]	Beijing V+/CHIN+	other	−	−

^a^[Z] = PZA DST, [R] = resistant, [S] = susceptible; ^b^ *M. tuberculosis* lineage according to MLPA; ^c^ A = very high confidence resistance mutations, C = mutations with an unclear role, D = mutations not involved in phenotypic resistance according to [10]

**Fig. 2:**
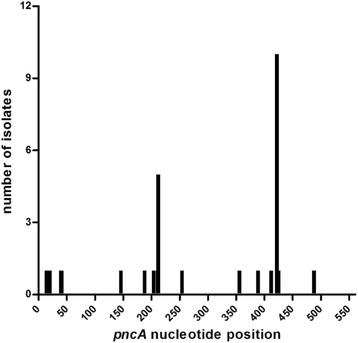
Distribution of *pncA* mutations identified in PZA resistant strains by sequencing and number of mutants per nucleotide polymorphisms

A mutation in *pncA* was found in all 33 isolates that were identified as PZA resistant by DST (Table 1). In total 18 different mutations were identified in the 33 PZA resistant strains, of which 17 were non-synonymous mutations and one strain was identified with a 1-bp insertion resulting in a frameshift. No *pncA* mutations were observed in 23 of 24 PZA susceptible strains. In one of the 24 PZA susceptible isolates a point mutation at amino acid position 16 (ATC > CTG) was identified leading to an I6L substitution. Of the 18 polymorphisms identified in *pncA* 16 have been described previously in the literature (Fig. 2 and Table 1).

**Fig. 3:**
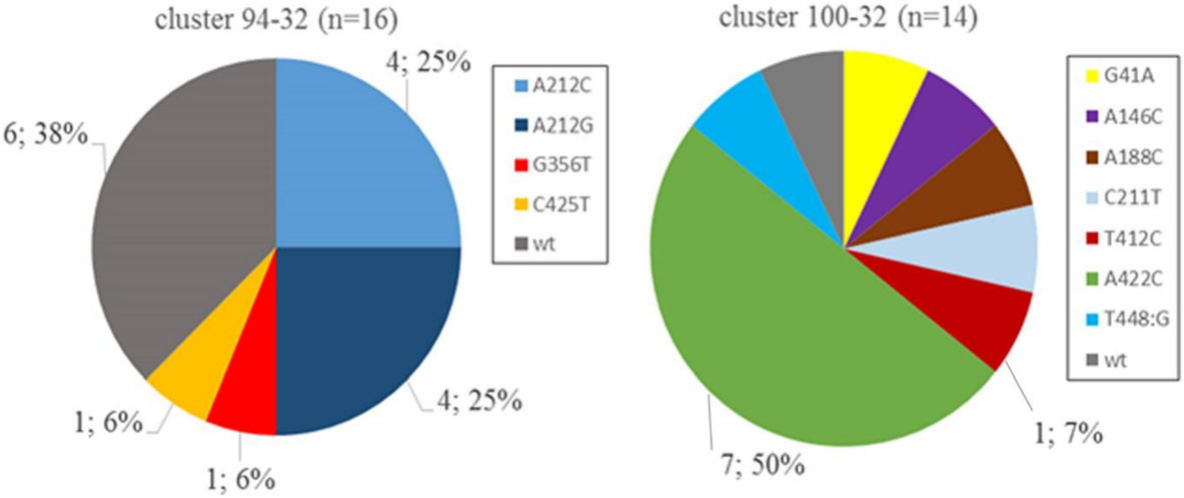
Distribution of *pncA* polymorphisms among the two largest MLVA clusters 94–32 and 100–32. Among all 57 strains with interpretable PZA DST results four clusters (94–32, 100–32, 407–32 and 1065–32) were identified. Clusters 407–32 and 1065–32 each consisted of two members, all carrying unique *pncA* mutations and are therefore not depicted in this figure. Distribution of strains with mutations in the *pncA* gene and their respective percentage in the cluster are listed. The legend indicates the position of the mutation in the *pncA* gene and the nucleotide change. In cluster 100–32 all but mutation A422C are present in individual isolates. No mutations are shared between the two clusters

Among the group of 57 strains with interpretable PZA DST results, four MLVA clusters were identified two of which contained members with different *pncA* mutations (Fig. 3 and Table 4). The largest cluster, MLVA type 94–32, consisted of 16 strains. Of these, 6/16 (37.5%) were PZA susceptible and had no mutation in *pncA*; eight strains were phenotypically PZA resistant and four had a codon 212 mutation leading to a CAT212CCT (4/16, 25.0%) and four a CAT212CGT (4/16, 25.0%) change. These eight isolates represented 45.0% (8/17) of MLVA 94–32 strains among all sequenced isolates. Two other mutations were identified within this cluster (TGG356 TTG, ACG425ATG) each in a single isolate (1/16, 6.2%).

The MLVA cluster 100–32 was formed by 14 isolates of which 13 were phenotypically resistant to PZA. One isolate was PZA susceptible by DST and did not carry a mutation in *pncA*. Seven of the 13 PZA resistant isolates (7/13, 53.8%) carried the same *pncA* mutation (CAG422CCG) and six isolates each (1/13, 7.8%) had different mutations in *pncA* (Table 1).

The CAG422CCG mutation resulting in a Gln141Pro substitution was observed in three MLVA types, 100–32, 100–35 and 9368–32 with the MLVA type 100–32 having the most representatives: this mutation was found in 50% (7/14) of all sequenced 100–32 strains.

MLVA clusters 407–32 and 1065–32 were both formed by two isolates each (Table 1 and Table 4). The strain carrying the I6L substitution (12–17,889) was one of two XDR-TB strains in the MLVA 1065–32 cluster. Unlike the 12–17,889 isolate the other member of the cluster, isolate 12–15,893, was PZA resistant by DST. PZA resistance was most likely conferred in 12–15,893 by a mutation in the *pncA* gene at position GTC20GCC leading to a V7A substitution. The I6L mutation, found in the isolate 12–17,889 has been previously reported in a large set of highly clustered isolates, called clade A, from Russia [26] together with four other characteristic genetic markers (*rpoB* E761D, P_eis_ c-37a, *pks15* T46I, Rv3254 P271P) [26]. In order to investigate whether these two isolates where part of the clade A cluster the genome sequences of isolates 12-15893 and 12-17889 were compared to the clade A strains from Russia. All five clade A specific markers were identified in the genome of strain 12–17,889, the isolate carrying the I6L mutation, but not in 12-15893. Genetic comparison revealed 43 SNPs difference between the two isolates (12-15893 and 12-17889) from this study and four SNPs difference between 12-17889 and the closest neighbour within the previously determined phylogeny of clade A (Fig. 2 in [26].

## Discussion

The high diversity of PZA resistance-conferring mutations reported in TB patient isolates as well as the absence of confidence in phenotypic testing challenge routine PZA resistance testing. In addition, it is unclear what clinical implications there are for the detection of PZA resistance in vitro. Although, as PZA resistance is associated with worse outcome, information on (the epidemiology of) PZA resistance will undoubtedly become increasingly important. Molecular methods aiming to identify resistance-conferring mutations in *pncA* seem more reliable in identifying PZA resistance than complex DST methods, as the majority of mutations observed in *pncA* have been shown to confer phenotypic resistance, albeit in varying levels [10, 11, 36, 37]. Reliable detection of PZA resistance even seems achievable using next generation sequencing with reported sensitivity of between 70.9% to 97% and a specificity of 94% [38, 39].

Sixteen of the 18 *pncA* polymorphisms identified in our study have been described previously [10] and 10/18 and categorized as very high confidence resistance mutations. In our collection of isolates the correlation between phenotypic testing and genetic analysis was very good; only one isolate that carried a mutation in the *pncA* gene, strain 12-17889, was susceptible to PZA by conventional DST. This finding was corroborated by a recent study where the *pncA*-I6L, mutation was also associated with susceptibility to PZA in a panel of clustered isolates [26] and was not categorised as a high confidence resistance mutation [10]. Interestingly, strain 12-17889 was tested phenotypically resistant by an alternative method for PZA DST not using low pH [40].

Three *pncA* mutations, A212G, A212C and A422C, were observed in multiple isolates. The mutations A212G and A422C were previously categorised as very high confidence resistance mutations ([10], Table 1). These mutations were not only present in closely related (Beijing) strains (MLVA 94–32 and 100–32) but were also identified in non-clustered (Beijing) strains. This may indicate a mild propensity to fix mutations in this particular locus for specific (Beijing) strains, a phenomenon called epistasis that we previously investigated but were unable to confirm for *rpoB* mutations [22]. The two clusters identified in our dataset (MLVA 94–32 and 100–32) are also known for their epidemic potential outside of Georgia [41–43].

**Fig. 4:**
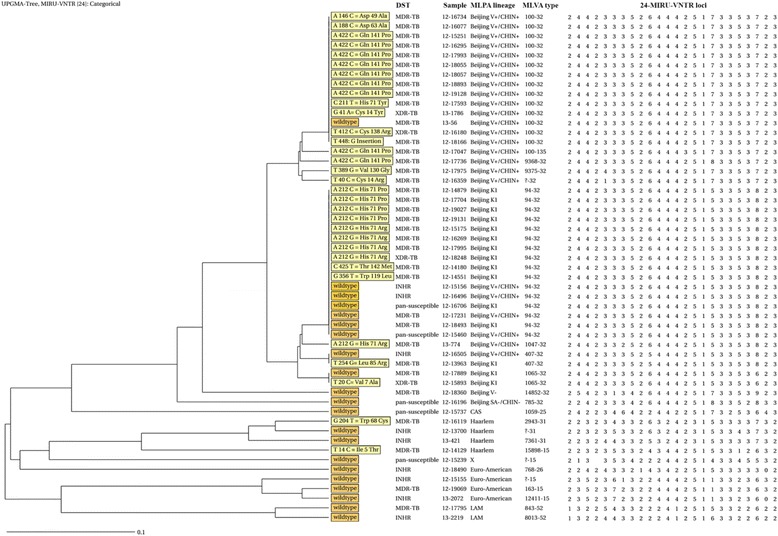
UPGMA-tree of isolates with a PZA DST profile. Of all 57 isolates with a PZA DST results, 53 isolates with a valid MIRU-VNTR profile were included

PZA resistance among new cases as well as clustering of PZA resistant strains with identical *pncA* mutations, as we found here (Table 1, Figs. 3 and 4), suggests that PZA resistant strains are transmissible. Contrary to the majority of first- and second line drug resistance mutations, multiple studies show that neither the fitness nor the virulence are significantly reduced by *pncA* mutations and data from Quebec moreover show that *pncA* mutants can maintain virulence and transmit [23, 44, 45]. However, as MIRU-VNTR typing has limited power to discriminate within genetically very similar strains only the comparison of the genomes of the respective strains can confirm our observation and rule out the possibility that the mutations in *pncA* have not been individually acquired.

Mutations that confer resistance to rifampicin or isoniazid and that result in fit transmissible bacterial strains are significantly more constrained. A very limited number of rare RIF and INH resistance mutations is found in genetically diverse clinical isolates. Consequentially, as genetically similar clinical isolates carry mostly identical MDR mutations (e.g. *katG*-S315 T and *rpoB*-S531 L) a proportion of isolates with identical MLVA types and these identical MDR mutations may be the result of independent mutational events rather than transmission of resistant strains. This is less likely for PZA where the available pool of fit mutations is much larger. Thus there might be a tendency to underestimate the de novo acquisition of rifampicin and isoniazid resistance. A recent study where longitudinal genomic comparison was performed supports the repeated acquisition theory for multiple drugs [25].

In addition to the large clusters, there were two smaller clusters in our collection, as identified by MLVA: 407–32 and 1065–32. Both clusters consisted of two members, all of which had acquired different *pncA* mutations, indicating independent events. One of the 1065–32 strains, 12-17889, carried an I6L mutation, which seems specific for a subgroup within the clade A MDR outbreak strain in the Samara region [26]. The other 1065–32 strain, 12–15,893, acquired resistance to PZA most likely via the V7A *pncA* mutation. Genomic comparison showed that there were only four SNPs difference between strain 12-17889 and the nearest neighbour of the clade A cluster, whereas there were 43 SNPs difference between strain 12–17,889 and strain 12-15893 indicating that they were much more distantly related.

The high prevalence of PZA resistant strains in Georgia, a highly endemic MDR(+) region, is a cause for concern as PZA resistance in MDR-TB cases is associated with unfavourable treatment outcomes. Bastos et al. showed that anti-TB treatment was more successful if patients infected with PZA susceptible MDR-TB received PZA, this association remained when they adjusted for fluoroquinolone and/or amikacin/kanamycin resistance [7]. Similar results were found in a study by Aung et al. showing that additional PZA resistance in the context of MDR-TB and fluoroquinolone resistance is associated with a worse treatment outcome [6]. In addition, PZA resistance may significantly jeopardise any PZA containing treatment regimens in the future. The sterilizing capacity of PZA as well as the synergistic action with drugs such as bedaquilline [3, 4] make it an attractive drug to include in both first- as well as second line treatment regimens.

This study has limitations. The isolates tested for PZA resistance were not specifically sampled for this study but were part of a previous study. We also did not sequence genes associated with PZA resistance other than *pncA.*

## Conclusions

We report an alarmingly high percentage of PZA resistance in MDR-TB isolates in Georgia. In this study we see evidence of both the transmission of *pncA* resistant strains as well as the acquisition of PZA resistance in the patient. This needs to be confirmed by whole genome sequencing of the respective strains and should also be extended to a larger study population ideally collected within a larger time period. Our data contribute to the rising pool of evidence showing the high incidence of PZA resistance among MDR-TB isolates. Health authorities and TB control programs should consider prospective genotyping and PZA testing to assure current effective MDR-TB treatment and to inform the design of new MDR-TB treatment trials. Knowledge of PZA resistance in MDR-TB strains is needed to ensure sufficient active drugs are provided to protect against the emergence of additional resistance, particularly against newly introduced drugs.

## Abbreviations

CTAB: cetylmethylammonium bromide
DST: drug susceptibility testing
INH: isoniazid
MDR-TB: multidrug-resistant tuberculosis
MGIT: mycobacteria growth indicator tube
MIRU-VNTR: mycobacterial interspersed repetitive unit-variable number of tandem repeats
MLVA: multiple locus variable number of tandem repeat analysis
Mtb: Mycobacterium tuberculosis
NRL: National Reference Laboratory
PZA: pyrazinamide
SNP: single nucleotide polymorphism
STR: streptomycin
TB: tuberculosis
WGS: whole genome sequencing
WHO: World Health Organization
XDR-TB: extensively drug-resistant tuberculosis
